# GPI-Anchored Protein Homolog *IcFBR1* Functions Directly in Morphological Development of *Isaria cicadae*

**DOI:** 10.3390/jof8111152

**Published:** 2022-10-31

**Authors:** Dong Li, Yunpeng Gai, Junlong Meng, Jingyu Liu, Weiming Cai, Fu-Cheng Lin, Hongkai Wang

**Affiliations:** 1 State Key Laboratory for Managing Biotic and Chemical Treats to the Quality and Safety of Agro-Products, Institute of Horticulture, Zhejiang Academy of Agricultural Sciences, Hangzhou 310021, China; 2 State Key Laboratory of Rice Biology, Institute of Biotechnology, Zhejiang University, Hangzhou 310058, China; 3 College of Food Science and Engineering, Shanxi Agricultural University, Taigu 030801, China; 4 School of Grassland Science, Beijing Forestry University, Beijing 100083, China; 5 State Key Laboratory for Managing Biotic and Chemical Treats to the Quality and Safety of Agro-Products, Institute of Plant Protection and Microbiology, Zhejiang Academy of Agricultural Sciences, Hangzhou 310021, China

**Keywords:** *Isaria cicadae*, synnemal formation, metabolites, nutrient utilization, transcriptome profile

## Abstract

*Isaria cicadae* is a famous edible and medicinal fungus in China and Asia. The molecular basis of morphogenesis and synnemal formation needs to be understood in more detail because this is the main source of biomass production in *I. cicadae*. In the present study, a fruiting body formation-related gene with a glycosylphosphatidylinositol (GPI) anchoring protein (GPI-Ap) gene homolog *IcFBR1* was identified by screening random insertion mutants. Targeted deletion of *IcFBR1* resulted in abnormal formation of synnemata, impairing aerial hyphae growth and sporulation. The IcFBR1 mutants were defective in the utilization of carbon sources with reduced polysaccharide contents and the regulation of amylase and protease activities. Transcriptome analysis of Δ*Icfbr1* showed that *IcFBR1* deletion influenced 49 gene ontology terms, including 23 biological processes, 9 molecular functions, and 14 cellular components. IcFBR1 is therefore necessary for regulating synnemal development, secondary metabolism, and nutrient utilization in this important edible and medicinal fungus. This is the first report illustrating that the function of IcFBR1 is associated with the synnemata in *I. cicadae*.

## 1. Introduction

*Isaria cicadae*, also called cicada flower in China, is an insect-derived fungus parasitic on cicada nymphs that has been used as food and herbal medicine for centuries [1]. Wild *I. cicadae* acts on a variety of diseases and is used to treat children with convulsions, children’s night terrors, palpitations, malaria, and so on, in the practice of traditional Chinese medicine [2]. In recent years, scientists have investigated various biological activities of *I. cicadae* and identified useful compounds, such as amino acids, cordycepin, polysaccharides, and mannitol, which are similar to compounds in *Cordyceps sinensis* [3]. Some substances are helpful to human health by regulating the immune system, activating macrophages [4,5], promoting phagocytosis of macrophages [6], improving the immune response of the liver, kidney, spleen, and thymus [7], having antiaging properties [8], protecting renal function; alleviating glomerulosclerosis and improving chronic renal failure [9,10], alleviating renal anemia, decreasing blood glucose [11], having antitumor properties [12], and improving vision [13].

*I. cicadae* can form a special type of asexual fruiting body referred to as coremia or synnema [4]. Mature synnemata are composed of large aggregations of mostly parallel hyphae to form a yellow-to-brown pigmented bundle of hyphae growing erect from the substrate. In *I. cicadae*, synnemata are the central part of this edible fungus with rich nutrition and clearly differentiated up to 3–8 cm high either in natural or artificial conditions. Synnemata have vigorous phospholipid metabolism, more curative metabolism, and a higher level of antioxidants and bioactive compounds such as HEA than other parts of *I. cicadae* [5]. Environmental conditions, including temperature and light, play a crucial role in synnemal formation [10]. To date, there is little known about molecular mechanisms involved in synnemal formation. In other fungi, such as *Aspergillus fumigatus*, many proteins are involved in fruiting body formation, including GPI proteins [14]. 

The glycosylphosphatidylinositol (GPI)-anchoring proteins (GPI-APS) are anchored to the plasma membrane and are responsible for producing linear polysaccharides to remodel the fungal cell wall [14,15]. In silico analysis revealed many putative GPI proteins in yeast and molds, some of which have major enzymatic functions in cell wall morphogenesis. There are 86 GPI proteins in the genome of *Aspergillus fumigatus*, and the functions of some GPI have been analyzed in detail. Most of the major GPI-APS exhibit enzymatic properties [16], such as proteases, chitosanase, glucanase, amylase, and transferases, which promote fungal morphogenesis through the remodeling of cell wall polymers [17]. Some GPI-APS are associated with cell wall morphology, being responsible for the extension of β-(1,3)-glucan, chitin–glucan linkages, and cell wall structures involved in adhesion and biofilm formation. GPI-anchored proteins with adhesive properties function directly in the formation of fungal fruiting bodies [15,18].

Recently, Labourel et al. found X325 protein, a new kind of GPI-AP with no enzymatic activity, through global transcriptome and secretome analyses. Their research showed that it is a membrane-bound protein with the C-terminal GPI anchor located at the periphery of the Hartig net [19]. The structure of Lbx325 displayed a fold similar to lytic polysaccharide monooxygenases (LPMOs), but Lbx325 extracted from fungal cell walls exhibited no enzymatic activity on plant cell wall components. The GPI anchor at the C terminus is present in Lbx325, but this feature is generally absent in fungal LPMOs. Labourel et al. discovered that Lbx325 represents a widespread copper-containing protein family in various fungal lineages, including saprotrophic and ectomycorrhizal fungi and yeast. These proteins do not perform oxidative cleavage of polysaccharides but have diverged in their functions in different fungal systems [19]. X325 protein is involved in fungal cell wall remodeling during Laccaria–Populus symbiosis [19].

The genes involved in fruiting body development in *I. cicadae* are unclear but determining them is necessary to establish the production of this medicinal fungus. Gene functions in *I. cicadae* can be studied thoroughly because a complete genome is available [20,21]. In this study, a fruiting body formation-related gene with a GPI-APS gene homolog was obtained by screening *Agrobacterium tumefaciens*-mediated transformation (ATMT) random insertion mutants. The insertion gene was determined by thermal asymmetric interlaced (TAIL)-PCR and named *IcFBR1*. The function was investigated in detail.

## 2. Materials and Methods

### 2.1. Fungal Strains, Vectors, and Culture Conditions

*Isaria cicadae* wild-type (WT) strain 2-2, stored at Zhejiang University, was cultured on PDA medium in a growth chamber at 25 °C with a 12 h light/12 h dark cycle. The vectors pBHt2, pCAMBIA1300, and pEC1 were stored in 20% glycerol at −80 °C. Extraction of plasmids was performed following the guidelines of Kits for Plasmid Extraction (Axygen, Hangzhou, China) after growing in LB medium with 100 ng/mL kanamycin at 37 °C, 150 rpm for 12 h.

### 2.2. ATMT Transformation and TAIL-PCR

The wild-type (WT) strain 2-2 of *I. cicadae* was used for random insertion mutant construction by plasmid pBHt2. The fungal transformation process followed the method of Chen et al. [22]. DNA flanking transfer DNA (T-DNA) was determined using the TAIL-PCR method according to the procedure by Mullins et al. [23].

### 2.3. Vector Construction and Verification of Mutant 

**Figure 1:**
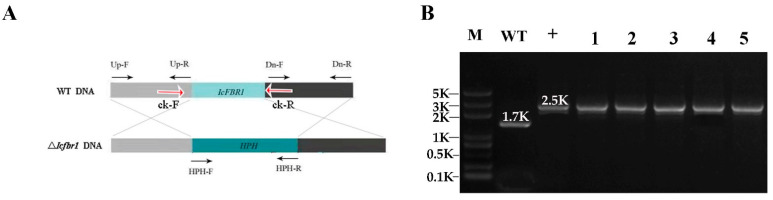
Identification of *IcFBR1* gene in *Isaria cicadae*. (**A**) Schematic diagram of targeted disruption of *IcFBR1* via homology-dependent gene replacement. (**B**) Verification of the null mutants by PCR. M—Trans 2K DNA marker; lane WT—wild-type strain 2-2; lane +—Knockout plasmid; lane 1–5—*IcFBR1* gene mutants.

The upstream and downstream 1.0–1.2 kb fragments of the *IcFBR1* gene were amplified with primers Up-F/Up-R and Dn-F/Dn-R from the wild-type strain genome. Using the plasmid pBHt2 as a template, the hygromycin B resistance gene *HPH* of about 1.4 kb was obtained using primers HPH-F and HPH-R. These three DNA fragments were fused to the *Hin*dIII and *Xho*I-linearized pCAMBIA1300 using a Fusion Enzyme Kit (Vazyme, Nanjing, China) (Figure 1A). The knockout cassette was transformed into the wild-type strain via *Agrobacterium tumefaciens*-mediated transformation (ATMT) according to the reported method [24,25]. Briefly, mutants were screened on a selective medium containing 350 µg/mL hygromycin B. Then, mutants were verified by PCR with primers ck-F/ck-R to determine whether *HPH* recombined into the *IcFBR1* deletion site (Figure 1B) to create a target gene knockout mutant. Finally, the insertion copies of *HPH* in the mutant were identified by Southern blotting following methods used by Chen et al. [22]. Briefly, 5 μg of genomic DNA of each sample was digested overnight at 37 °C with *Xba*I and *Hin*dIII, respectively. Digested DNA fragments were separated by Electrophoresis in 1% agarose gel and blotted onto nylon membrane Hybond-N^+^ (Amersham Pharmacia Biotech, Amersham, UK). Using the knockout vector as a template, a 1065 bp DNA fragment containing a 633 bp partial genomic DNA and a 432 bp partial HPH gene was amplified with primers S-F/S-R and used as a probe template. The probe was labeled using the DIG High Prime DNA Labeling and Detection Starter Kit II (Roche, Mannheim, Germany) described in the manufacturer’s manual. A single copy of knockout mutants was also confirmed by the Q-PCR method according to the assay described by Lu et al. [24]. Briefly, when tubulin is the reference gene, a single copy of the target gene was determined as ∆∆CT = 0.9~1.3, where ∆∆CT = (CT_HPH_ – CT_tubulin-m_) – (CT_gene_ – CT_tubulin-w_), CT_HPH_ is the CT value of HPH in the mutant, CT_tubulin-m_ is the CT value of tubulin in the mutant, CT_gene_ is the CT value of target gene in the wild-type strain, and CT_tubulin-w_ is the CT value of tubulin in the wild-type strain.

For the complementation experiments, a 2.5 kb native *IcFBR1* gene (containing a promoter and coding sequence (CDS), but no termination codon) was cloned from the *I. cicadae* genome by primers FBR1-CF/FBR1-CR and inserted into the *Pst*I-linearized vector pEC_1_. pEC_1_ was derived from pCAMBIA1300 containing a neomycin gene (*G418*) and green fluorescent protein gene (*GFP*). The complementary vector was transformed into the mutant ∆*Icfbr1* via ATMT. The complementary mutants of ∆*Icfbr1*-c were selected on a selection medium containing 1100 μg/mL neomycin and confirmed by Southern blotting. All primers used in this paper are listed in Table S1 (Supporting Material).

### 2.4. Effects of IcFBR1 on Nutrition Utilization and Phenotypic Characterization

For the fungal growth assay, the WT, ∆*Icfbr1*, and the complimentary strain ∆*Icfbr1*-c were cultured on 7 cm diameter plates containing 17.5 mL PDA medium at 25 °C for 10 days. To investigate germination of conidia, 20 μL of the spore suspensions (1 × 10^5^ conidia/mL in liquid PDB medium) was separately smeared on PDA medium and water agar, respectively, and incubated at 25 °C for 24 h.

The utilization of carbon sources affected by *IcFBR1* was as follows: single 1% carbon sources (glucose, sucrose, fructose, lactose, maltose, soluble starch, sodium carboxymethyl cellulose, ethanol, or glycerol) were separately configured in minimal media (MM) [NaNO_3_ 6 g/L, KCl 0.52 g/L, MgSO_4_·7H_2_O 0.52 g/L, KH_2_PO_4_ 0.25 g/L, Agar 15 g/L]. A piece of the colony of each strain tested was incubated on 7 cm plates containing 17.5 mL configured medium at 25 °C with a 12 h/12 h light/dark cycle for 10 days. Stress tests were performed as follows: A drop of the spore suspensions (30 μL) was dripped onto separate PDA plates with 0.01% sodium dodecyl sulfate (SDS), 200 μg/mL Calcofluor white (CFW), or 200 μg/mL Congo red, and incubated at 25 °C in the dark for 7 days. All experiments were performed at least twice, with three replicates of each treatment.

### 2.5. Observation of Fluorescence Fusion Proteins in I. cicadae and Quantitative Real-Time PCR

GFP expression of the complementary strain ∆*Icfbr1*-c was observed using a fluorescence microscope (Filter: FITC and TRITC) (Nikon Eclipse 80i, Nikon, Japan) after culturing on a PDA plate or rice substrate according to conditions described in Section 2.8. In order to ensure the reliability of the results, RT-PCR was also performed. Total RNAs were extracted from spores and mycelia with Trizol following the manufacturer’s procedure (TaKaRa Bio, Beijing, China). Reverse transcription of cDNA was conducted using the PrimeScript^TM^ RT reagent kit with gDNA Eraser (TaKaRa, Japan). Expression of *IcFBR1* was performed on a Real-Time PCR Detection System Mastercycler (Eppendorf, Germany) with an SYBR Premix Ex Taq kit (Tli RNaseH Plus, TaKaRa Bio, Beijing, China) according to the manufacturer’s instructions. Tubulin was selected as the reference gene, and *IcFBR1* gene expression was calculated using 2^−^^∆CT^, where ∆CT = CT_gene_ – CT_Tubulin_. The primers used in this experiment are listed in Table S1 (Supporting Material).

### 2.6. Lipid Droplet Stain with BODIPY

BODIPY was dissolved to 1 μg/mL with 2% DMSO for lipid droplet staining. The conidia were stained by BODIPY solution for 5 min, then observed by fluorescence microscopy. 

### 2.7. Assay of Determination of Polysaccharide and Enzyme Activity

A total of 10^5^ spores of each strain tested were transferred to a 500 mL flask containing 200 mL potato dextrose broth, incubated at 25 °C while shaking at 150 rpm for 7 days. The mycelia of each strain were harvested by filtering with 3 layers of gauze and washed with distilled water 3 times. Subsequently, the mycelia were dried by using the freeze dryer. The total polysaccharide content of mycelia was determined using the phenol sulfuric acid method, according to Zhang et al. [26]. The activities of enzymes were investigated by inoculating a 0.5 cm diameter of mycelium on an enzyme-induced medium and incubating at 25 °C in the dark for 7 d to observe the enzymatic hydrolysis cycle. Amylase was assayed on a medium with 1% starch, 1.5% agar, and 0.1% potassium iodide solution. Protease was assayed on a medium containing 1% milk and 1.5% agar. Chitinase was detected on medium with 1.5 × 10^−4^ g/L Bromocresol purple, 0.03% MgSO_4_•7H_2_O, 0.3% (NH_4_)_2_SO_4_, 0.2% KH_2_PO_4_, 0.1% citric acid, 0.45% colloidal chitin, and 200 μL/L Tween-80.

### 2.8. Effects of IcFBR1 on the Growth and Formation of Synnema

The wild-type strain 2-2, knockout mutant ∆*Icfbr1,* and the complementary strain ∆*Icfbr1-c* were cultured on PDA medium in a growth chamber at 25 °C with a 12 h light/12 h dark cycle for phenotypic observation. Synnema was induced in rice or flour medium, according to previous research [21,27]. Briefly, fungal strains were inoculated on rice or flour medium in darkness at 25 °C for one week, then transferred to 25 °C with a day/night cycle of 12 h light/12 h darkness for two weeks to form synnemata.

### 2.9. Transcriptome Analysis

Mycelia of strains tested were cultured on rice medium at 25 °C for 7 days, harvested, and ground into a fine powder in liquid nitrogen using a mortar and pestle for RNA isolation. Total RNA was extracted using Trizol, according to the manufacturer’s instructions (TaKaRa, Japan). Then, the NovaSeq 6000 system was used for transcriptome sequencing. RNA-Seq was performed using three biological replicates of each sample. Fast QC was used to detect the quality of transcriptome data [28]. Trimmomatic was used to compare transcriptome data after removing low-mass bases [29]. HISAT was used to compare transcriptome data to the reference genome of WT CCAD02. Htseq-count was used to count the number of genes [30]. DEseq2 (R2) was used to screen differentially expressed genes (DEGs) with a preset threshold of *p* < 0.05 and log_2_FC (fold change) > 1 [31]. Blast2 was used to annotate the Gene Ontology (GO) of DEGs [32]. Enrichment analysis of GO annotation results was carried out using WEGO online tool [33]. Differences in KEGG pathway analysis were performed with the KEGG database (https://www.genome.jp/kaas-bin/kaas_main, accessed on 13 July 2022) [34].

### 2.10. Statistical Analysis

The significance of treatments was determined by DPS 9.5 software, and means were separated by a least significant difference (LSD) test (*p* ≤ 0.05).

## 3. Results

### 3.1. The IcFBR1 Gene Screening in I. cicadae

A random insertion mutation library was constructed with plasmid pHt2 by ATMT in *I. cicadae*. Six mutants were obtained with reduced aerial hyphae growth compared with the phenotype of WT strain 2-2. Southern blot confirmed that single-copy insertion induced the change in hyphae growth in these mutants. The gene location of the genomic insertion in mutant ChGD was screened using the TAIL-PCR method, and a 260 bp DNA fragment was obtained after sequencing. BLAST research in the Genbank database showed that the gene fragment had 90% homology with *Isaria fumosorosea* ARSEF 2679 hypothetical protein (ISF_08023, GenBank accession No.: XM_018851626). After searching the genome of *I. cicadae* (GenBank accession no. MWMN00000000), we obtained its homologous gene. Since it is a fruiting body-related gene, we named it *IcFBR1*. The sequence length of the *IcFBR1* gene is 916 bp, putatively encoding 235 amino acids (Supporting Information Supplementary File S1). Amino acid BLAST using NCBI showed more than 50% identity with a putative GPI-AP in the fungal genomes KAF5707377 of *Fusarium mundagurra*, XP_008598424 of *Beauveria bassian*a, TQV90811 of *Cordyceps javanica*, OAQ78299 of *Purpureocillium lilacinum*, and OAQ58852 of *Pochonia chlamydosporia*. Conserved domains search in the GenBank dataset showed that it belongs to the lytic polysaccharide monooxygenase (LPMO) auxiliary-like protein (Supporting Material Figure S1A). Members of this family are related to LPMOs but have no enzymatic activity in the oxidative cleavage of polysaccharides [19]. Predicting structure analysis indicated that this protein has two transmembrane structures (Supporting Material Figure S1B). GPI prediction analysis showed a GPI-anchor signal sequence at C-terminal, and there is a signal peptide at the N-terminal composed of 17 amino acids, MKLNLIISLALAGLAAA (http://gpi.unibe.ch/, accessed on 13 July 2022).

### 3.2. Validation of Gene Knockout Mutants

The mutant ∆*Icfbr1* was obtained by homologous recombination. The primers ck-F/ck-R were used to detect whether *HPH* replaced *IcFBR1*. The bands of ∆*Icfbr1* were different from that of WT, but the same as that of the control (pK-hph) (Figure 1B), demonstrating that the *IcFBR1* gene was successfully replaced by the HPH gene. Moreover, we used Southern blotting to detect the gene copy number and showed that the mutant ∆*Icfbr1* and wild-type strain 2-2 both had one band with different sizes (Supporting Material Figure S2). We select NO. 2 and NO. 5 mutants as single-copy mutants and use mutant NO. 2 for further research. Single-copy knockout mutants were also confirmed by Q-PCR in that the ∆∆CT value is 1.1 and 1.2, respectively. According to Lu et al. [24], this means that the gene is a single copy. The results above indicated that the strains gained in this experiment were consistent with the expected results but also indicated that the HPH gene exactly substituted for the *IcFBR1* gene.

### 3.3. IcFBR1 Is Involved in the Development of I. cicadae

The complementary strain ∆*Icfbr1*-c was gained by transforming the complementary vector with fused *IcFBR1* and GFP gene into knockout mutant ∆*Icfbr1*.

**Figure 2:**
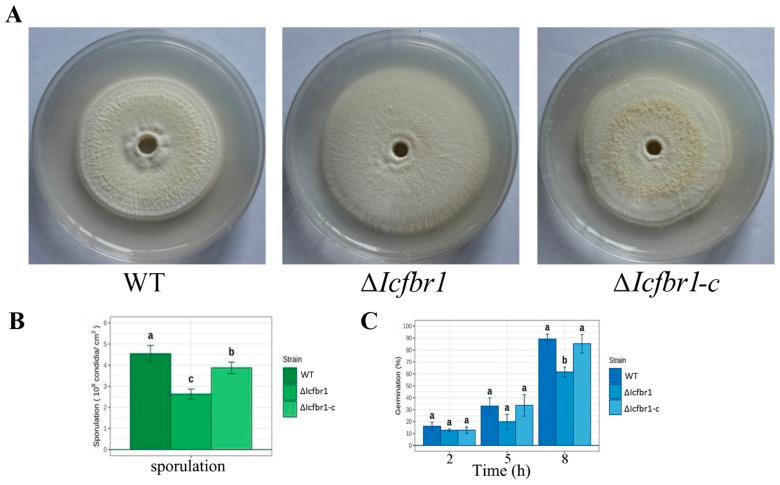
Phenotype characters of *IcFBR1* null mutant. (**A**) Colony of WT, ∆*Icfbr1*, and ∆*Icfbr1-c* were grown on PDA medium at 25 °C for 10 days and photographed. (**B**) Sporulation on PDA. (**C**) Spore germination on water agar. The same letters in the same stress item indicate a nonsignificant difference estimated (*p* ≤ 0.05). All tests were repeated at least twice with three replicates of each treatment.

To investigate the possible effect of *IcFBR1* on colony growth, sporulation, and conidia germination, the mutants and wild-type strain were cultured on PDA at 25 °C for 10 days. After measuring, we found that the colony diameters of ∆*Icfbr1* were larger than the WT and the complementary ∆*Icfbr1*-c (*p* < 0.05) (Figure 2A), but the hyphae were very sparse and short, and the colony thickness of ∆*Icfbr1* were thinner than the wild-type strain about 3 times (Supporting Material Figure S3). Compared with the wild type, sporulation in ∆*Icfbr1* was significantly diminished by almost twofold (Figure 2B). When spores germinated in a water agar medium, the germination rate of ∆*Icfbr1* was slower than the wild-type strain at 8 h (*p* < 0.01) (Figure 2C), with the same tendency on PDA (Supporting Material Figure S4). The results showed that the *IcFBR1* gene is crucial for the colony growth and sporulation development of *I. cicadae*.

### 3.4. IcFBR1 Is Involved in the Utilization of Carbon Sources by I. cicadae

**Figure 3:**
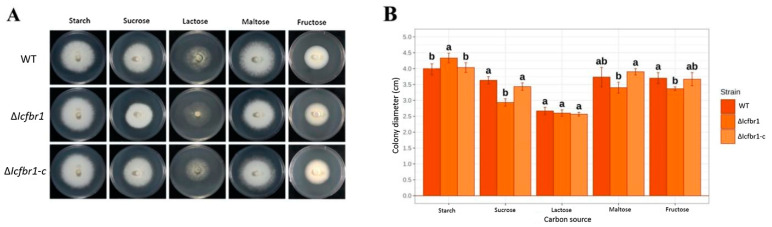
Colony morphology of wild type, ∆*Icfbr1,* and ∆*Icfbr1-c* were grown on minimal media (MM) with one type of carbon source. (**A**) Colony of wild type, ∆*Icfbr1*, and ∆*Icfbr1-c* were grown on minimal media (MM) with starch, sucrose, lactose, maltose, and fructose, respectively, at 25 °C with a 12 h/12 h light/dark cycle for 10 days. (**B**) Colony diameter statistics for (**A**). The same letters in the same stress item indicate a nonsignificant difference estimated (*p* ≤ 0.05). All tests were repeated at least twice with three replicates of each treatment.

To measure whether *IcFBR1* affects the utilization of carbon sources, fresh colonies of the wild-type strain, ∆*Icfbr1*, and ∆*Icfbr1*-c were inoculated on MM media added with different carbon sources and incubated at 25 °C, with 12 h light/12 h dark cycles for 7 days. The morphological characteristics and colony diameters are shown in Figure 3A. After measuring the colony diameters, we found that on the flour medium, the growth of ∆*Icfbr1* was faster than the wild type and complementary ∆*Icfbr1*-c (*p* < 0.05). Measuring the colony diameters on media with sucrose, maltose, and fructose, we found that the growth of ∆*Icfbr1* was slower than the wild-type strain and complementary strain ∆*Icfbr1*-c (*p* < 0.05). On the lactose medium, the colony diameters of the three strains had no differences, but the aerial hyphae of ∆*Icfbr1* were significantly reduced compared with the WT and complementary ∆*Icfbr1*-c. In other media, there was no obvious difference (Figure 3B). The results suggested that the *IcFBR1* gene may influence the utilization of partial carbon sources in *I. cicadae*.

### 3.5. IcFBR1 Has No Effect on the Integrity of the Cell Wall of I. cicadae

When spores were inoculated on different cell wall stress media for 7 days, there was no significant difference in the growth of the WT, ∆*Icfbr1*, or complementary ∆*Icfbr1-c* (Supporting Material Figure S5), indicating that *IcFBR1* does not affect cell wall integrity.

### 3.6. Expression of GFP-Tagged IcFBR1 in Different Stages of I. cicadae Life Cycle

**Figure 4:**
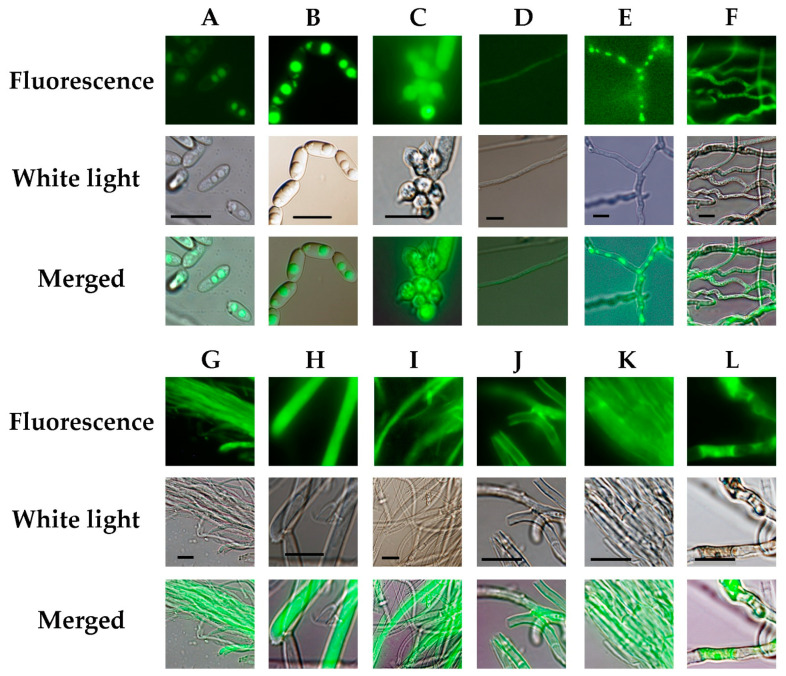
Microscopical analysis of GFP-tagged IcFBR1 in *I. cicadae* strain ∆*Icfbr1-c*. (**A**) Spore staining by BODIPY. (**B**–**E**) GFP expression when cultured on PDA. (**B**) Spores; (**C**) Conidiophore and phialides; (**D**) Young hyphae, very faint GFP expressed in young hypha cells; (**E**) Old hyphae; (**L**): GFP expression when cultured on rice media. (**F**) Hyphae in young synnemata cultured 8 days after inoculation (DAI); (**G**) GFP expression in synnemal cell with parallel young hyphae; (**H**) Synnemal cells cultured 10 DAI (young synnema); (**I**) Hyphae cultured 14 DAI. (**J**) Synnemal cells (mature synnema) cultured 18 DAI, GFP expression on the cell wall. (**K**) Synnemal cells (mature synnema) cultured 21 DAI, GFP expression on cell wall and septa. (**L**) GFP expressed in mature cells growing from the outside surface of synnema. Bar = 10 μm.

Observation of the GFP fluorescence of the spores and hyphae of the complementary strain ∆*Icfbr1*-c under the microscope indicated that *IcFBR1* was expressed in cells of hyphae, phialides, and conidia (Figure 4B–E) when incubated on PDA. GFP was highly expressed in a type of globular structure in mature conidia but was very faint in young conidia (Figure 4B). To confirm if these globular structures are lipid droplets, conidia of the wild-type strain were stained with BODIPY and observed by fluorescence microscopy. The results showed that the globular structures in mature conidia are lipid droplets (Figure 4A). These results illustrated that the IcFBR1 protein was located on lipid droplets in mature conidia. GFP expression in phialides was very strong (Figure 4C). In hyphae, GFP was highly expressed in the condensed protoplast structure of mature cells (curved hyphae cells) and cells of conidiophore but was very faint in young cells in hyphae (Figure 4D–F). To investigate the GFP expression on synnemata, a hand section was made and observed under a fluorescence microscope (Figure 4F–L). GFP was highly expressed in young synnemal cells compared with the mature synnemal cells (Figure 4H,I), but GFP can be observed at the surface of mature synnemal cell wall, especially at the sites of branch and septa (Figure 4J,K). Other cells of highly expressed GFP are mature cells growing from the outside surface of synnema (Figure 4L). These results indicate that IcFBR1 proteins are located in mature cells and production structures. 

**Figure 5:**
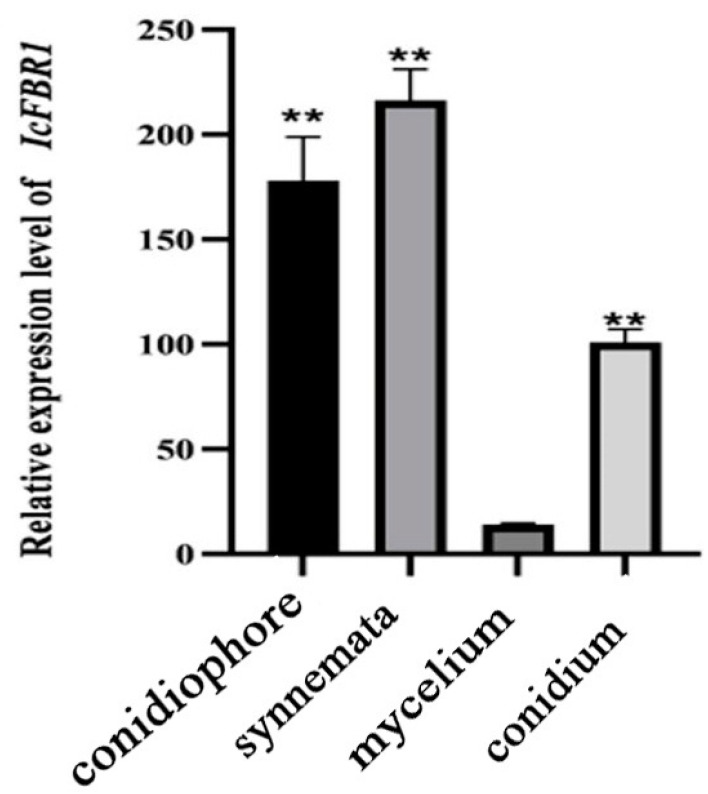
Expression of *IcFBR1* gene in *I. cicadae* wild-type strain. Gene expression of *IcFBR1* in a different part of *I. cicadiae*. “**” indicate a significant difference at *p* < 0.01. All tests were repeated at least twice with three replicates of each treatment.

The RNA of spores, synnemata, and hyphae of the wild-type strain were extracted, and RT-PCR verification was conducted after reverse transcription. Compared with the internal reference gene tubulin, the expression of the *IcFBR1* gene in the spore and synnemata was much higher than that in the hyphae (Figure 5). There are 17.7, 13.2, and 8.2 times more *IcFBR1* gene expressions in synnemata, conidiophore, and conidium than in mycelium, respectively. 

### 3.7. Effects of IcFBR1 on I. cicadae Key Metabolites

Polysaccharides, cordycepin, and adenosine are important active substances of *I. cicadae*. In the present research, we found there are no effects on the contents of cordycepin and adenosine, but the contents of polysaccharides in ∆*Icfbr1* were reduced when compared with the wild-type strain and ∆*Icfbr1-c.* The polysaccharide content of mutant ∆*Icfbr1* was 2.84%, less than 3.91% in the wild-type strain and 3.87% in ∆Icfbr1-c. It suggested that IcFBR1 had an effect on the contents of polysaccharides.

We compared the activities of amylase, protease, and chitinase of the wild type, ∆*Icfbr1*, and complementary ∆*Icfbr1-c*. Our results showed that the hydrolytic circle of ∆*Icfbr1* in the starch medium was significantly larger (*p* < 0.01) than that of the wild type (1.84 ± 0.09 cm of ∆*Icfbr1* compared with 1.33 ± 0.05 cm of the wild-type strain 2-2) and complementary ∆*Icfbr1*-c, but the hydrolytic circle of the ∆*Icfbr1* in milk powder medium was significantly smaller than that of the WT and complementary ∆*Icfbr1*-c (0.97 ± 0.05 cm diameter of ∆*Icfbr1* compared with 1.20 ± 0.00 cm of wild the wild-type 2-2 and 1.18 ± 0.02 cm of ∆*Icfbr1*-c) (*p* < 0.01). There was no significant difference in the diameter of the hydrolytic circle on the chitin medium (1.67 ± 0.05 cm diameter of ∆*Icfbr1* *and* wild-type strain 2-2) (Supporting Material Figure S6). The results showed that *IcFBR1* inhibits the activity of amylase in *I. cicadae* and has no effect on the synthesis of chitinase but promotes the synthesis of protease. The main components of the insect epidermis are protein and chitin, 70% of which is protein, meaning that protein is the main component of the insect epidermis. Secreting a large amount of protease is very important in the process of entomopathogenic fungi infecting insects [35]. Our result infers that the protease function of *IcFBR1* may be essential in pathogenicity to the host.

### 3.8. IcFBR1 Is Involved in the Formation of Synnemata

**Figure 6:**
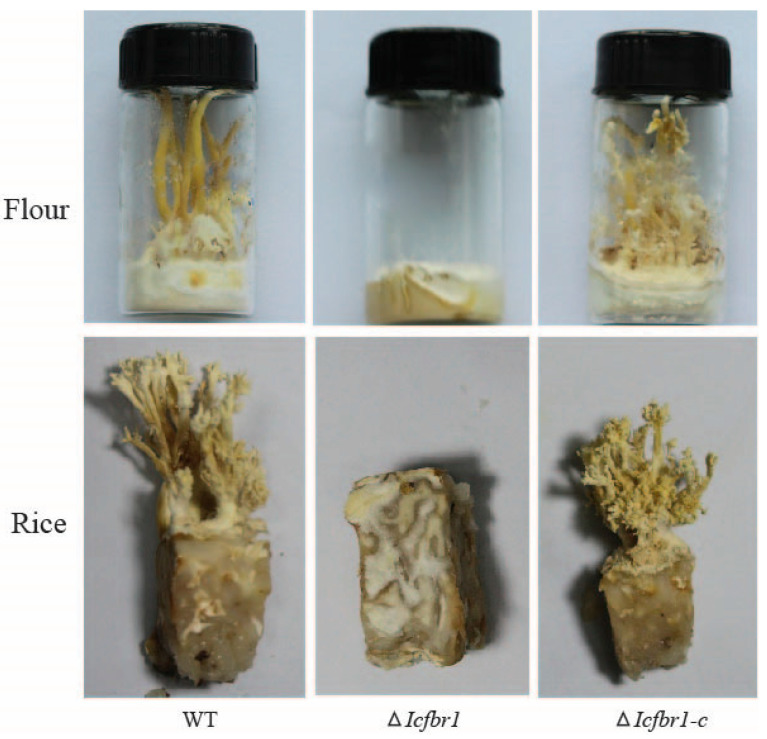
Production of synnemata of WT, ∆*Icfbr1*, and ∆*Icfbr1-c* on flour and rice substrates.

Synnemata are the main parts of this kind of fungus that people process and eat; therefore, whether synnemata can be produced is extremely important for cultivating edible fungi. To check if synnemal formation was affected by *IcFBR1*, the wild-type strain, ∆*Icfbr1*, and complementary ∆*Icfbr1-c* were incubated on flour and rice culture. In wild-type strain 2-2, some mycelium curled up and aggregated to form hyphal knots (Supporting Material Figure S7) after vegetative mycelium covers the surface of the substrates. A large number of mycelia then grow in parallel and adhere together to form synnemata from the hyphal knots. However, ∆*Icfbr1* did not produce synnemata even though there was no hyphal knots formation on the surface of the substrate. The results indicated that the *IcFBR1* gene had a strong effect on the formation of the synnemata (Figure 6); *IcFBR1* gene defection can block the synnemal formation at the primary synnemata differentiation stage.

### 3.9. Transcriptome Analysis

**Figure 7:**
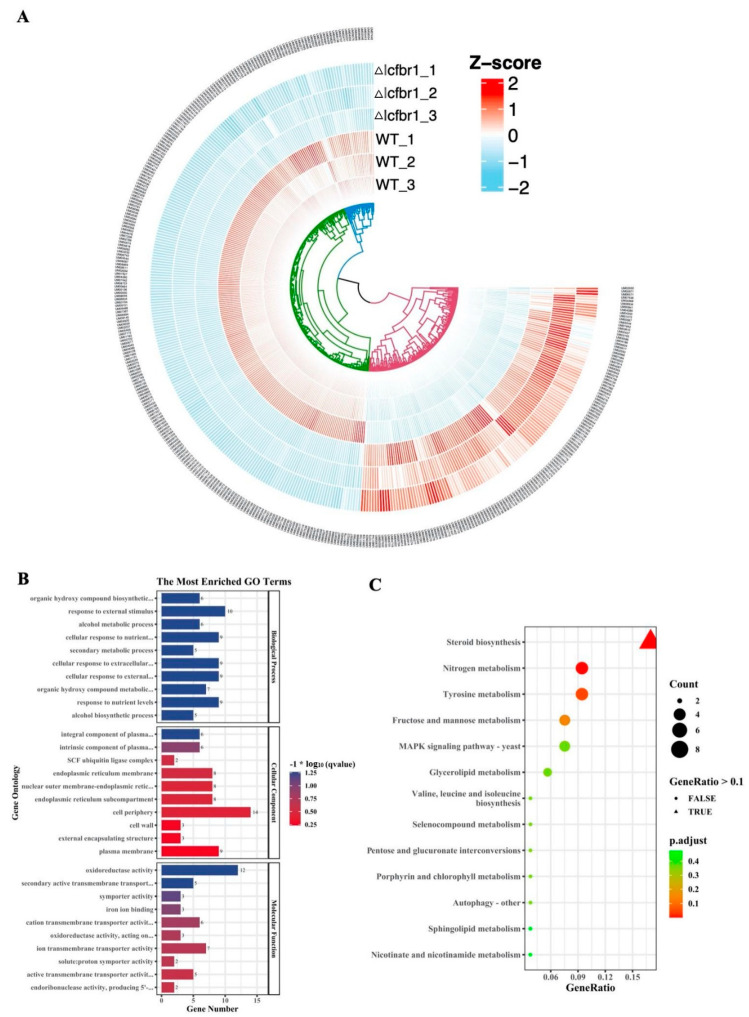
Transcriptome analysis of differentially expressed genes (DEGs) in Δ*Icfbr1*. (**A**) Heatmap of RNA-Seq expression z-scores computed for genes that are differentially expressed (p adj < 0.05, |log_2_(foldchange)| > 1) between ∆*Icfbr1* and wild type; (**B**) Gene Ontology (GO) enrichment analysis of DEGs between ∆*Icfbr1* and WT. The X-axis represents the number of genes in a category, and the Y-axis represents the GO term; (**C**) Scatter plots of KEGG pathway enrichment statistics based on a statistical analysis of DEGs in ∆*Icfbr1*.

A total of 21,701,446 raw reads were obtained by sequencing the total RNA, and 21,700,017 clean reads were retained in mutant ∆Icfbr1. Moreover, a total of 23,137,568 raw reads were obtained by sequencing the total RNA in the wild-type strain, and 23,136,222 clean reads were retained after trimming the low-quality bases. The clean reads were mapped to the reference genome of *I. cicadae* 2-2 and showed that 89.73% of ∆Icfbr1 RNA-Seq reads were mapped to the genome of *I. cicadae* 2-2, and 90.73% of wild-type reads were mapped to the genome of *I. cicadae* 2-2. In total, 378 differentially expressed genes, including 131 upregulated genes and 247 downregulated genes, were identified in the IcFBR1 mutant compared with the *I. cicadae* wild type (Figure 7A).

GO enrichment analysis (Figure 7B and Supporting Material Figure S8) revealed that inactivation of IcFBR1 significantly affected the expression of many genes enriched in molecular function (MF), including “oxidoreductase activity (12)”, “secondary active transmembrane transporter activity (5)”, “symporter activity (3)”, “iron ion binding (3)”, “cation transmembrane transporter activity (6)”, “oxidoreductase activity, acting on paired donors, with incorporation or reduction of molecular oxygen (3)”, “ion transmembrane transporter activity (7)”, “solute:protonsymporter activity (2)”, “active transmembrane transporter activity (5)”, “endoribonuclease activity, producing 5’-phosphomonoesters (2)”; biological process (BP), including “organic hydroxy compound biosynthetic process (6)”, “response to external stimulus (10)”, “alcohol metabolic process (6)”, “cellular response to nutrient levels (9)”, “secondary metabolic process (5)”, “cellular response to extracellular stimulus (9)”, “cellular response to external stimulus (9)”, “organic hydroxy compound metabolic process (7)”, “response to nutrient levels (9)”, “alcohol biosynthetic process (5)”; and cellular component (CC), including “integral component of plasma membrane (6)”, “intrinsic component of plasma membrane (6)”, “SCF ubiquitin ligase complex (2)”, “endoplasmic reticulum membrane (8)”, “nuclear outer membrane-endoplasmic reticulum membrane network (8)”, “endoplasmic reticulum subcompartment (8)”, “cell periphery (14)”, “cell wall (3)”, “external encapsulating structure (3)”, and “plasma membrane (9)” (Supporting Material Tables S2–S5). 

KEGG enrichment analysis (Figure 7C and Supporting Material Figure S8C) revealed that inactivation of IcFBR1 significantly affected the expression of many genes enriched in “steroid biosynthesis (9)”, “nitrogen metabolism (5)”, “tyrosine metabolism (5)”, “fructose and mannose metabolism (4)”, “valine, leucine and isoleucine biosynthesis (2)”, “selenocompound metabolism (2)”, “glycerolipid metabolism (3)”, “MAPK signaling pathway (4)”, “pentose and glucuronateinterconversions (2)”, “porphyrin and chlorophyll metabolism (2)”, “autophagy—other (2)”, “sphingolipid metabolism (2)”, “nicotinate and nicotinamide metabolism (2)”, “dNAreplication (2)”, “mitophagy (2)”, “lysine degradation (2)”, “autophagy (3)”, and “meiosis (3)” (Supporting Material Tables S2–S5).

We found there were some important genes significantly downregulated in mutant ∆Icfbr1. Results of GO enrichment analysis showed that the inactivation of IcFBR1 can significantly downregulate eight genes in response to external stimulus, including UM04610 homolog (peptide synthetase, Log_2_FC = −1.06), UM06847 (ERG1, squalene, Log_2_FC = −1.40), UM05243 homolog (UME6, transcriptional regulatory protein pro1, Log_2_FC = −1.11), and UM01375 homolog (HXT5, belongs to the major facilitator superfamily. Sugar transporter (TC 2.A.1.1) family, Log_2_FC = −1.00); 5 genes in cation transmembrane transporter activity (molecular function, including UM04089 homolog (CTR3, ctr copper transporter family protein, Log_2_FC = −1.17)), UM01375 homolog (HXT5, belongs to the major facilitator superfamily; sugar transporter (TC 2.A.1.1) family, Log_2_FC = −1.00) and P22189 homolog (Calcium-transporting ATPase, Log_2_FC = −1.48); 7 genes in transmembrane transporter activity (molecular function, GO:0022857, *p* value = 0.0237508213537631), including UM04089 (CTR3, ctr copper transporter family protein, Log_2_FC = −1.17), UM01375 (HXT5, belongs to the major facilitator superfamily. Sugar transporter (TC 2.A.1.1) family, Log_2_FC = −1.00), UM02277 (c4-dicarboxylate transporter malic acid transport protein, Log_2_FC = −1.43). The functions of these transmembrane transporters also involve an integral component of the plasma membrane, response to nutrient levels, and cellular response to an extracellular stimulus; 10 genes in the cell periphery (cellular component, GO:0071944, *p* value = 0.0403767506382644), including UM04089 (CTR3, ctr copper transporter family protein, Log_2_FC = −1.17), UM01375 (HXT5, belongs to the major facilitator superfamily; sugar transporter (TC 2.A.1.1) UM09662 (GDP GTP exchange factor Sec2p, Log_2_FC = −1.08), etc. (Supporting Material Table S3). 

The results KEGG enrichment analysis of the down-regulated genes showed that the inactivation of IcFBR1 can significantly down-regulate 3 genes in nitrogen metabolism (ko00910), including the UM00349 homolog (nitrate reductase is a key enzyme involved in the first step of nitrate assimilation in plants, Log_2_FC, −2.55); 2 genes in tyrosine metabolism (ko00350), including the UM07748 homolog (TCRP, 4-hydroxyphenylpyruvate dioxygenase, Log_2_FC, −1.63); 2 genes in MAPK signaling pathway in yeast (ko04011), including the UM07739 homolog (protein tyrosine kinase, Log_2_FC, −1.11), and UM08477 homolog (CAT1, occurs in almost all aerobically respiring organisms and serves to protect cells from the toxic effects of hydrogen peroxide, Log_2_FC, −1.01); 3 genes in autophagy in yeast (ko04138), including the UM05243 homolog (UME6, transcriptional regulatory protein pro1, Log_2_FC, −1.11); and 3 genes in meiosis in yeast (ko04113), including the UM01375 homolog (HXT5, belongs to the major facilitator superfamily. Sugar transporter (TC 2.A.1.1) family, Log_2_FC, −1.00), etc. (Supporting Material Table S4).

## 4. Discussion

Entomopathogenic fungi, such as *Cordyceps sinensis*, have been used for medicinal purposes for several centuries, particularly in China, Japan, and other Asian countries [36]. As an important member of the Cordyceps family, *I. cicadae* has attracted research attention and has been utilized in traditional medicine for a long time [10]. Functional analysis of numerous genes in *C. militaris* has been conducted in recent years, including *CmWC-1* [37], *CmVVD* [38], *CmSnf1* [39], and so on. In contrast, there are few reports on gene functions in *I. cicadae*. In this study, we found that *IcFBR1* affects the growth and development of *I. cicadae* and, most critically, that it may function directly in the formation of synnemata. For *I. cicadae*, growing hyphae in the early period and producing synnemata in the late period are extremely critical steps. Results in the present study indicated that *IcFBR1*gene could influence differentiation at the early stage of synnemal formation. This is the first report in which the gene function is related to synnemata (coremium) growth. 

In general, LPMOs are copper-dependent enzymes that degrade polysaccharides through an oxidative mechanism. Recently, a new family of proteins (X325) with the LPMO fold having a His brace structure overturned the accepted thinking within the field of polysaccharide degradation and illustrated that these proteins are not LPMOs [19]. Reports indicated that the proteins in this family might regulate fungal growth in *P. anserine* [19] and control hyphal cell–cell fusion and maintenance of mutualistic interaction in an *Epichloë festucae* [40] and ryegrass system, as well as copper acquisition in *Cyptococcus neoformans* [41]. In the present study, a conserved domain search of *IcFBR1* in GenBank showed that this gene has an LPMO-auxiliary-like domain shared with X325 of *Laetisaria arvalis*. Loss-of-function of *IcFBR1* resulted in defects in coremium formation and reduction in conidial formation, but no phenotype of “crippled growth”, “impaired for cell-cell fusion”, or “effect of pathogenicity” [19]. Our results provide new evidence that this new protein family can regulate synnemata development in *I. cicadae*. The mechanism of *IcFBR1* in regulating development will be further studied in the future.

Many genes play important roles in the formation and development of fruiting bodies, and their absence will have adverse effects on fruiting body development, even resulting in no fruiting bodies. For example, *exg1* is involved in fruiting body development in *Lentinula edodes* [42], and *FruA* is an essential transcription factor for fruiting body development in *Myxococcus xanthus* [43]. In *Flammulina velutipes*, *fv-pda* is specifically expressed through the entirety of fruiting body development, and the transcript is abundant despite mature fruiting bodies. This suggests that chitin deacetylase (*CDA*) functions directly in the process of fruiting in *F. velutipes* [44]. In the present study, our results indicate that *IcFBR1* regulated the colony growth, development of sporulation and synnemal formation, and secondary metabolism of *I. cicadae*. By observing the fluorescence, IcFBR1 can be basically localized on the plasma membrane but can localize at the inner surface of the cell wall in the mature synnemata, which is consistent with the structural analysis that this kind of protein has a GPI domain. Post-translation regulation may result in a variety of locations in different morphogenesis stages. Numerous biological processes were affected by the disruption of *IcFBR1*, as illustrated by the transcriptome analyzed in this research. Some genes involved in cellular components composing cell structures, including external encAPSulating structure, cell wall, cell periphery, and plasma membrane, are absent or significantly downregulated in the *IcFBR1* mutant, and these components are very important for fungal growth and differentiation. Through analysis of the transcriptome, we found that *IcFBR1* seriously influenced the steroid biosynthesis pathway. Some genes involved in the MAPK signal pathway and calcium-transporting pathway were sharply downregulated when *IcFBR1* defected. The roles of the signal pathway in synnemata formation need to be investigated in the future. The ability to produce synnemata is extremely important in artificial breeding, and ∆*Icfbr1* produced no synnemata under the same conditions. Therefore, we think *IcFBR1* is very important to *I. cicadae*.

In general, simple multicellularity in fungi is typically involved in linear processes such as vegetative mycelia, in which all cells are in direct contact with the external environment to absorb nutrients and O_2_, while complex multicellularity is defined as three-dimensional differentiated structure formation with a spatially and temporally integrated developmental program. In PDA and other media plates, *I. cicadae* grow only in a simple multicellular manner in which the growing hyphae rarely adhere to each other, and no synnemata are produced. In artificial substrates, however, such as rice and certain culture conditions, a large number of conidia are produced after vegetative hyphae cover the surface of the substrates. Then some hyphae begin to curl up and aggregate to form the primary hyphal knot. Synnemata grow up erect to the substrate along with the extension of primary hyphal knots. A large number of conidiophores and conidia are then produced on the surface of the synnemal stalks. Considering that the genetic mechanisms of synnemata are not yet fully understood, *I. cicadae* may be an excellent model to study the development of complex multicellularity since simple and complex multicellularity coexist in this species under certain conditions. In this study, high expression of *IcFBR1* in curved hyphae cells indicates that the *IcFBR1* gene may be involved in the formation of hyphal knots, the preliminary structure of synnemata. Mutants with a defective *IcFBR1* gene exhibited a simple multicellular stage (clonal formation in plates) that can undergo morphogenesis but failed in synnema formation (complex multicellular stage), indicating that the growth and development mechanisms of synnemata differ from vegetative mycelium and conidial sporulation on PDA plates, despite the fact that both are known as asexual development stages in *I. cicadae*.

## 5. Conclusions

A gene encoding GPI-APS homologous protein (IcFBR1) was cloned, and its functions were investigated in the present study in *I. cicadae*. We found that *IcFBR1* regulates the formation of synnemata, hypha growth, sporulation, biosynthesis of polysaccharides, the enzyme activity of amylase and protease, and utilization of carbon sources in this important edible and medicinal fungus. This is the first report illustrating the roles of *IcFBR1* in synnemata formation, fungal growth, sporulation, and polysaccharides biosynthesis in filamentous fungi, and it provides a foundation for future research.

## Data Availability

The transcriptome project has been deposited at NCBI BioProject under the accession PRJNA865918 (https://www.ncbi.nlm.nih.gov/bioproject/PRJNA865918, accessed on 13 July 2022). All gene sequences, genome, gff3 file, proteomes, gene ontology (GO), COG category, CAZy, KEGG, PFAMs, NR, and eggNOG annotation of Cordyceps cicadae 2-2 for bioinformatic analysis are available in the Ze-nodo repository at https://doi.org/10.5281/zenodo.6963421, accessed on 13 July 2022. The high-resolution figures and gene expression patterns of each GO term and KEGG pathway are available on the figshare repository: https://doi.org/10.6084/m9.figshare.20436168.v1, accessed on 13 July 2022.

## Supplementary Materials

The following supporting information can be downloaded at: https://www.mdpi.com/article/10.3390/jof8111152/s1, Figure S1: analysis of *IcFBR1* functional domain, Figure S2: Southern blot of T-DNA insertion mutants and *Icfbr1* null mutant ∆*Icfbr1*, Figure S3: colony thickness of gene mutant and the wild-type strain, Figure S4: spore germination rate of *I. cicadae* on PDA, Figure S5: growth of strains of *I. cicadae* inoculated on different cell wall stress media incubated at 25 °C in the dark for 7 days, Figure S6: hydrolytic circle of strains of *I. cicadae* on the different medium for enzyme activity test, Figure S7: morphology of hyphal knot effected by *IcFBR1* Gene, Figure S8: (A) gene ontology (GO) hierarchical graph analysis of the DEGs: biological process (BP), (B) gene ontology (GO) hierarchical graph analysis of the DEGs: molecular function (MF), (C) gene ontology (GO) hierarchical graph analysis of the DEGs. Cellular Component (CC), File S1: DNA sequences of IcFBR1 and amino acid sequence encoded by the target gene, Table S1: oligonucleotide primers used in this study, Table S2: the differentially expressed genes (DEGs) between IcFBR1 mutant and *Isaria cicadae* wild type, Table S3: GO enrichment analysis of the differentially expressed genes (DEGs) between IcFBR1 mutant and *Isaria cicadae* wild type, Table S4: KEGG enrichment analysis of the differentially expressed genes (DEGs) between IcFBR1 mutant and *Isaria cicadae* wild type, Table S5: protein sequence and it NR functional annotation.
