# Supplementary material for: GPI-Anchored Protein Homolog IcFBR1 Functions Directly in Morphological Development of Isaria cicadae
Source: J Fungi (Basel). 2022 Oct 31;8(11):1152. doi: 10.3390/jof8111152 (PMC9695133; doi:10.3390/jof8111152)
Supplement: Supplementary file 1 [file jof-08-01152-s001.zip › Supplementary Figures.pdf]

Supplementary Figures

A

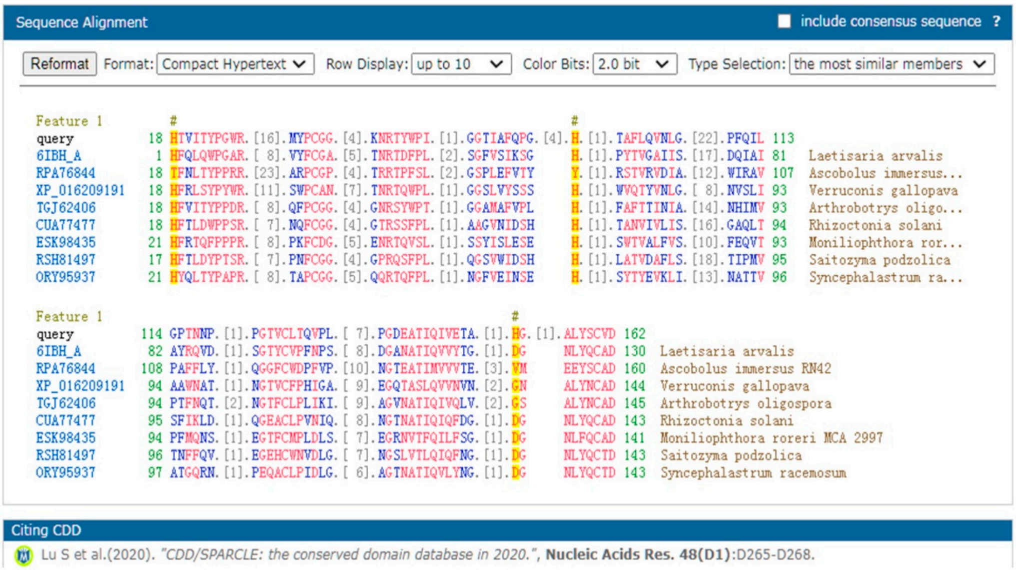

B

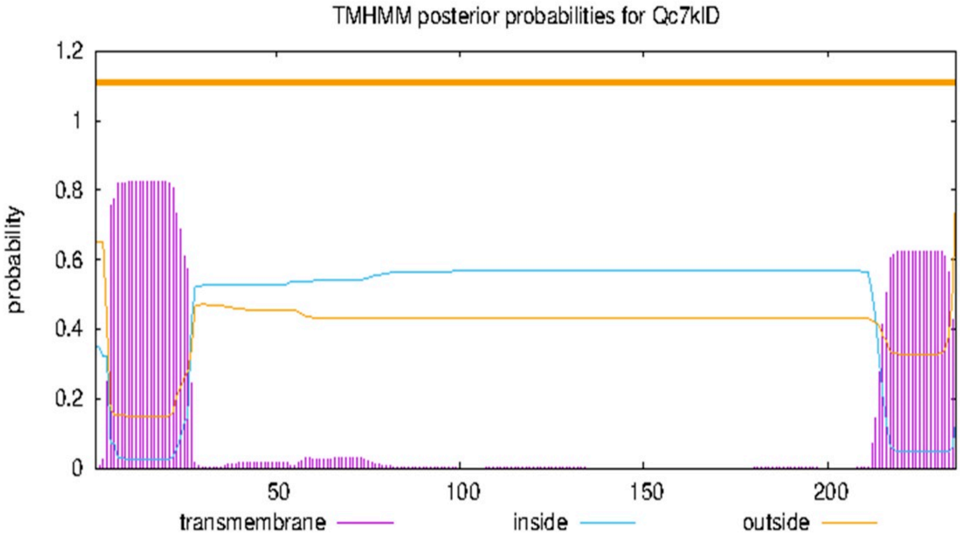

Fig. S1 Analysis of *IcFBR1* functional domain.

A. Searching result of Conserved domains of *IcFBR1* in the GenBank dataset. Amino acids sequences of LPMO auxiliary-like domain in different organisms. 6IBH_A refers to X325 of *Laetisaria arvalis.*

B. Prediction of transmembrane structures of IcFBR1. The Prediction was performed through (https://www.novopro.cn/tools/)

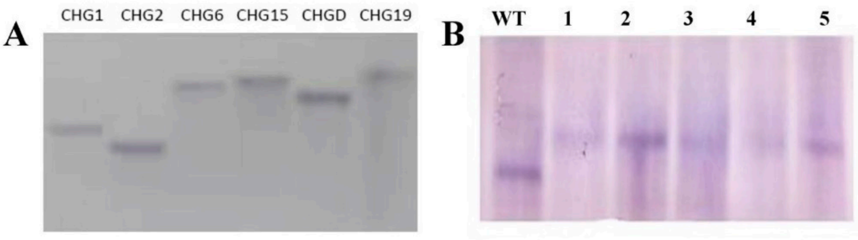

Fig. S2 Southern blot of T-DNA insertion mutants and *Icfbr1* null mutant Δ*Icfbr1*.
A. verificationof T-DNA insertion mutants. Lane CHGD indicates the target mutant.
B. Gene copy number tested by southern blot in mutant Δ*Icfbr1*. Lane WT: wild-type strain. Lane 1-5: different null mutants.

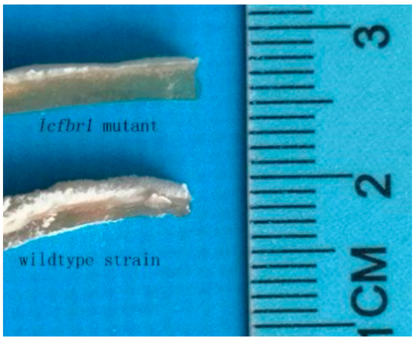

Fig. S3 colony thickness of gene mutant and wild-type strain.
A piece of colony cut of and side placed to show colony thickness of mutant Δ*Icfbr1* and wild type strain 2-2 incubated on PDA at 25℃for 10 days.
Icfbr1 mutant: Δ*Icfbr1*, Wildtype strain: 2-2. The colony thicker of 2-2 is much more than 3 times of Δ*Icfbr1*.

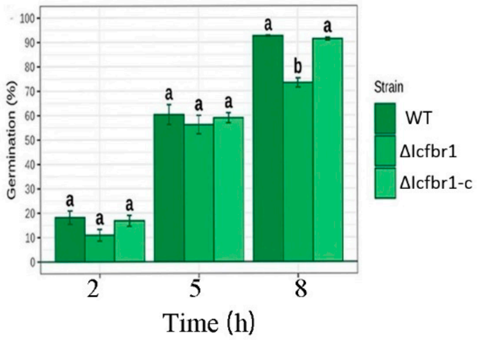

Fig. S4 Spore germination rate of *I. cicadae* on PDA.

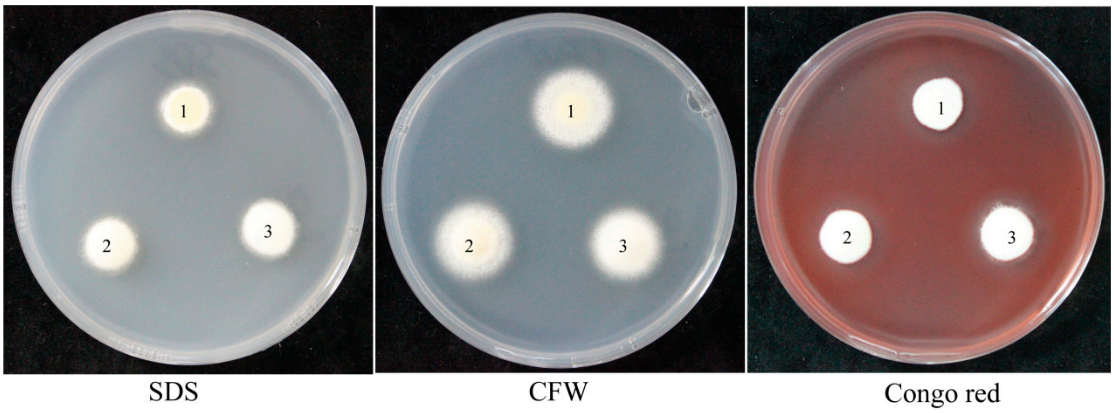

Fig. S5 Growth of strains of *I. cicadae* inoculated on different cell wall stress media incubated at 25 °C in the dark for 7 days. 30μL spore suspensions were dripped onto separate PDA plates contained with 0.01% sodium dodecyl sulfate (SDS), 200 μ g/ml Calcofluor white (CFW), or 200 μ g/ml Congored, respectively.

1: wild type 2-2, 2: complementary Δ*Icfbr1-c*, 3: Δ*Icfbr1*.

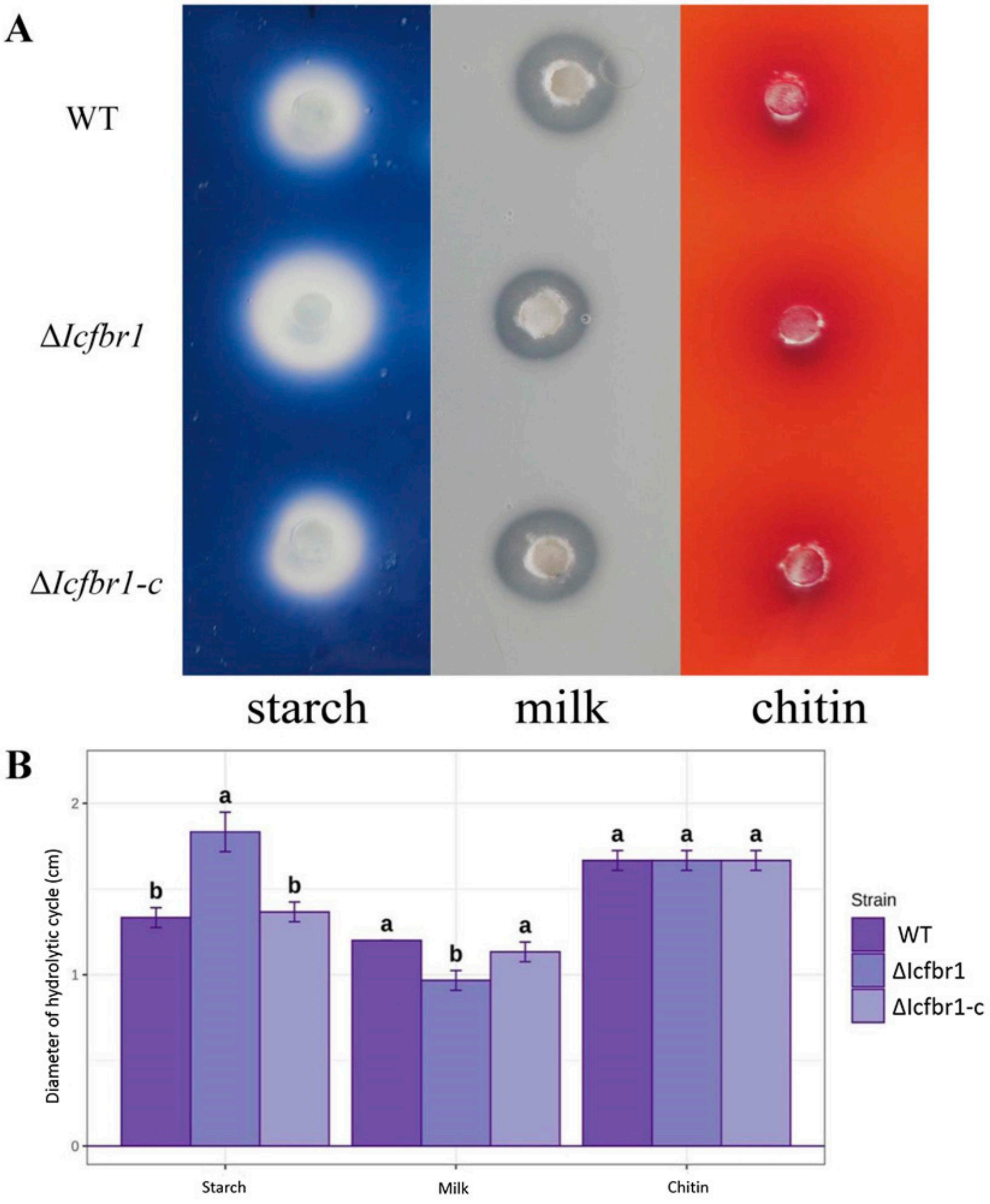

Fig. S6 Hydrolytic circle of strains of *I. cicadae* on the different medium for enzyme activity test. A. Pictures of different enzyme hydrolytic circles. B. Diagram of the diameter of the Hydrolytic circle of different strains.

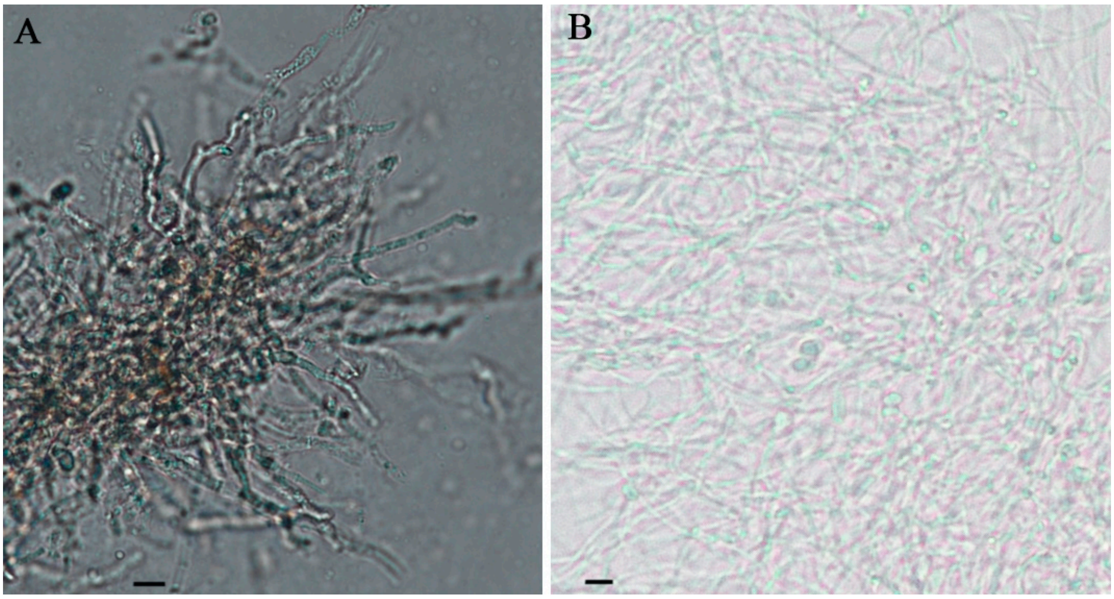

Fig. S7 Morphology of hyphal knot effected by *IcFBR1* Gene.

A. Morphology of hyphal knot of wild-type strain 2-2. Hyphae associated within hyphal knot are twisted, hyphal cells are shorten, diameter become thick, branches, tangled together, the cells of the central part are nearly spherical, cell wall thickness. B. No hyphal knot formation by mutant Δ*Icfbr1*. The mycelia is loose although the colony is velvet. The diameter of hypha cells is thinner than that of hypha knot.

A

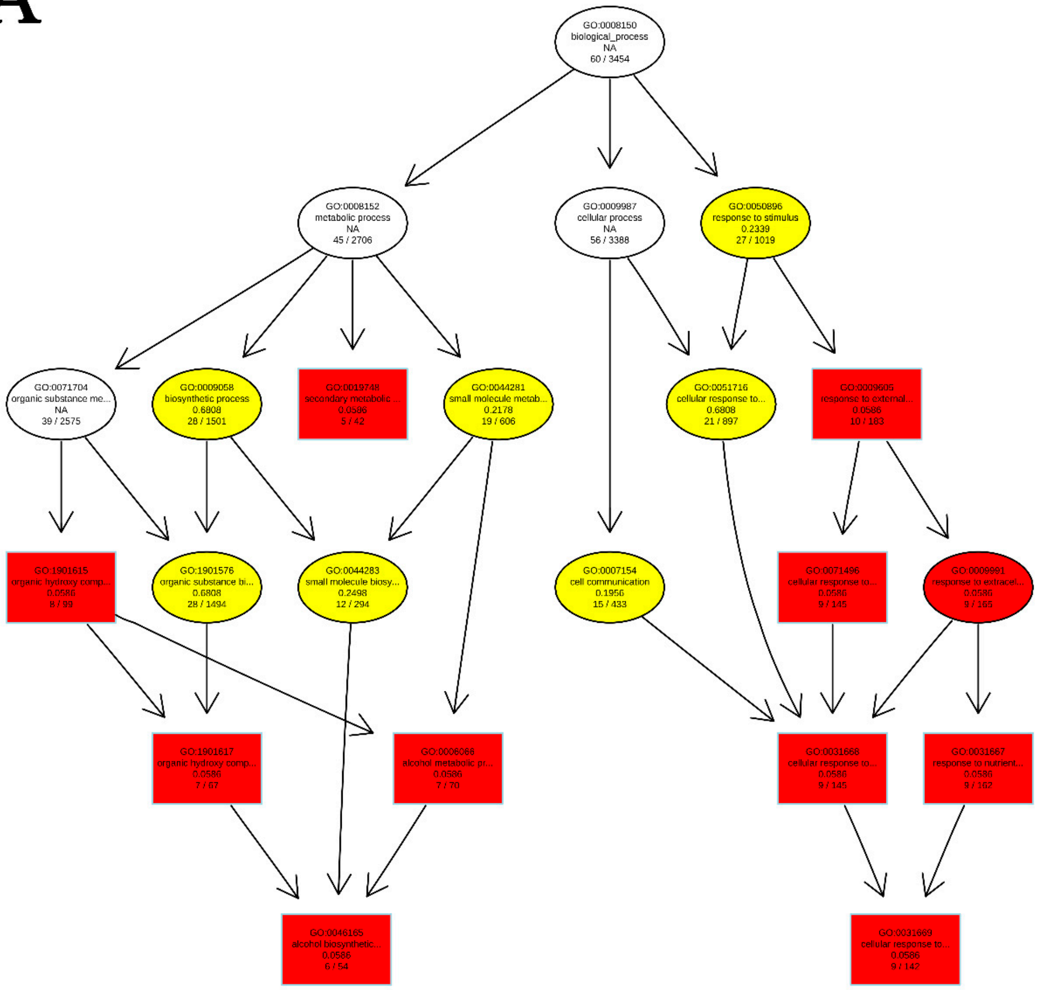

Fig. S8 A Gene Ontology (GO) hierarchical graph analysis of the DEGs: Biological Process (BP)

B

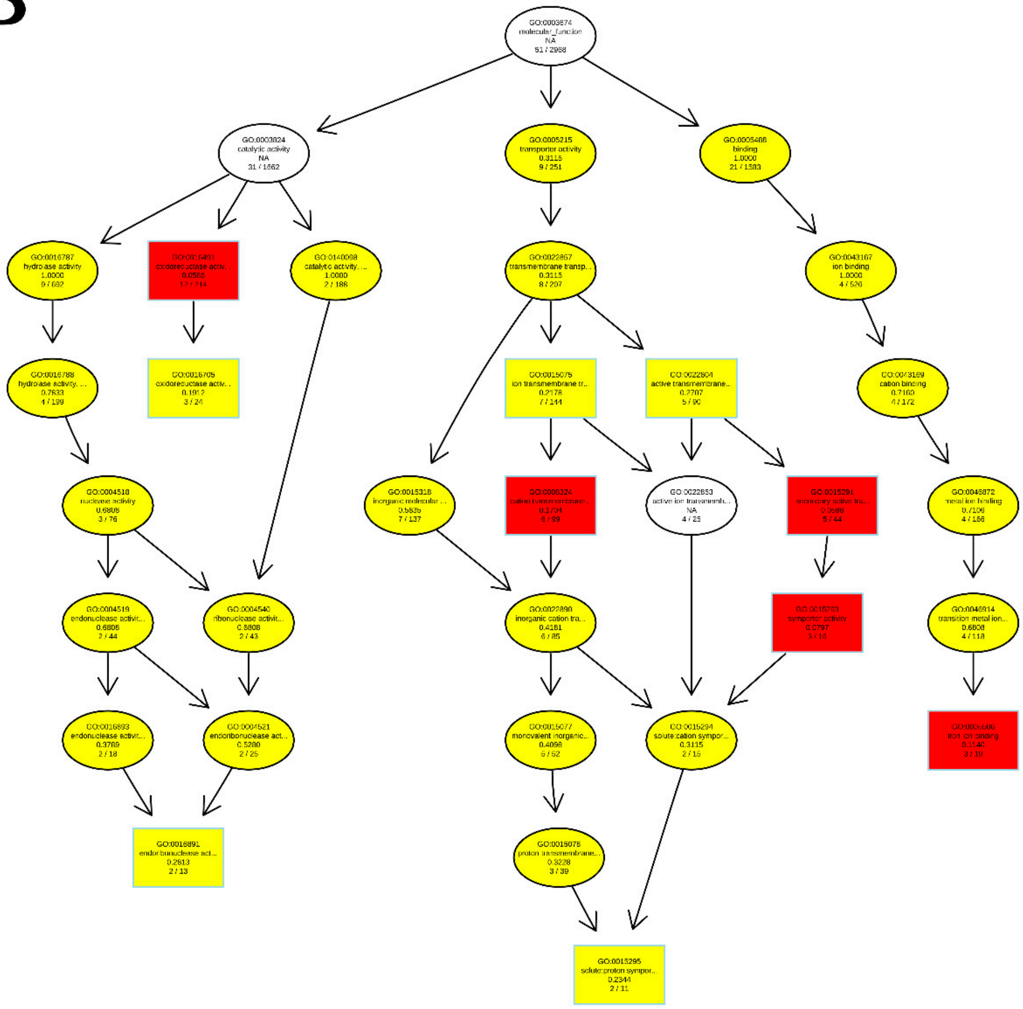

Fig. S8 B Gene Ontology (GO) hierarchical graph analysis of the DEGs: Molecular Function (MF)

C

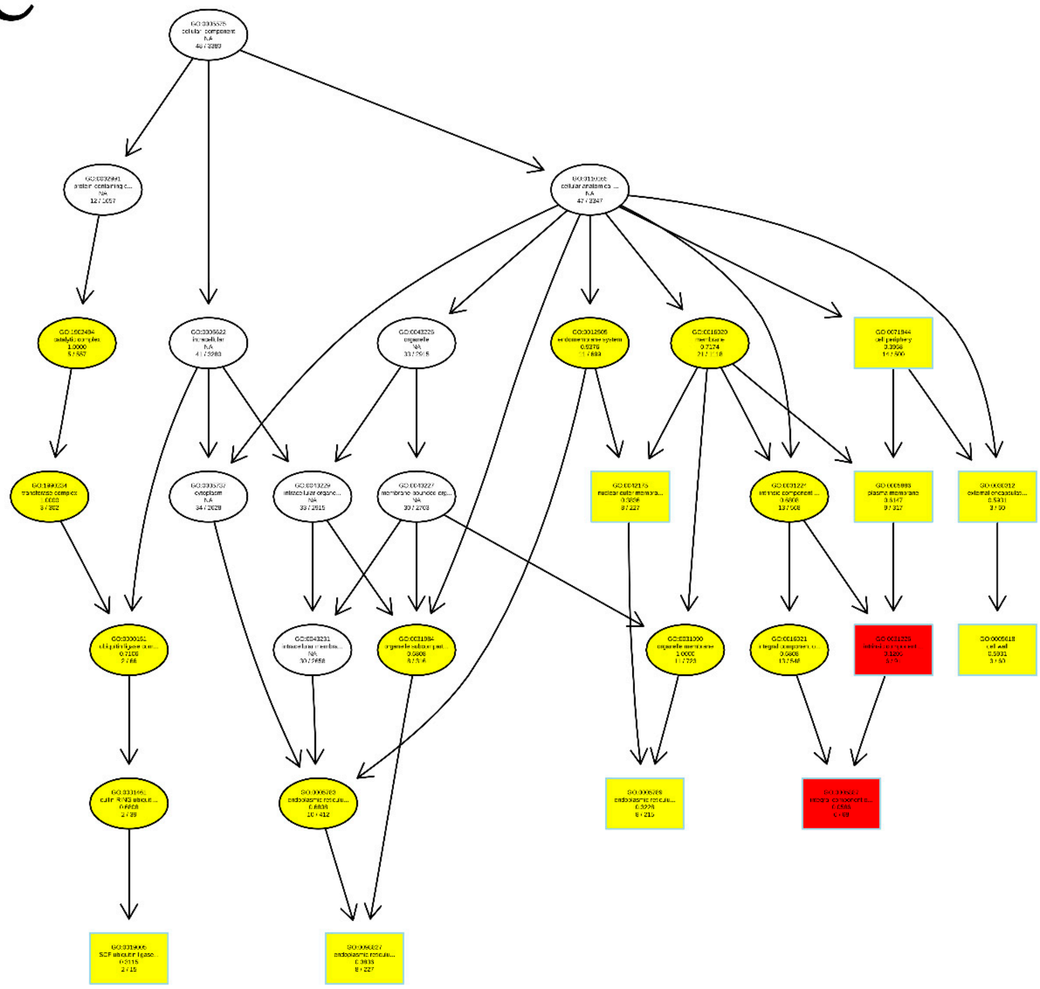

Fig. S8 C Gene Ontology (GO) hierarchical graph analysis of the DEGs. Cellular Component (CC).

Fig. S8 Gene Ontology (GO) hierarchical graph analysis of the DEGs. A: Biological Process (BP), B: Molecular Function (MF), C: Cellular Component (CC).
