# Supplementary material for: GPI-Anchored Protein Homolog IcFBR1 Functions Directly in Morphological Development of Isaria cicadae
Source: J Fungi (Basel). 2022 Oct 31;8(11):1152. doi: 10.3390/jof8111152 (PMC9695133; doi:10.3390/jof8111152)
Supplement: Supplementary file 1 [file jof-08-01152-s001.zip › Supplementary File S1.pdf]

Supplementary File S1: DNA sequences of *IcFBR1* and amino acid sequence encoded by the target gene

>DNA sequence of *IcFBR1* gene in *Isaria cicadae*

ATGAAGCTCAACTTGATCATCAGCCTCGCCCTCGCCGGCCTGGCCGCCGCACACACCGTCATCTCCTACCCG
GGCTGGCGCGGTAACAACCTCATCACCAACGACACCTTTCCCTACGGCATGCAATGGATGTATCCCTGTAAG
TGTTTTGTCCTCGCCGCGCTCGACGTCGGGCCGCCGCGCTCCTCCTTCTTACCACGCTCCTCCTTCTCACTGC
GCTTCCCCCTCGCCACTCAATTCCCTTGCTGACCATCAACTTCCCAGGCGGCGGCATGGGCGTCACCAAGAA
CCGCACCTACTGGCCCATCGGCGGCGGCACCGTCGCCTTCCAGCCCGGCTGGTTCCGCGGCCACTCCCTTG
CCTTCGTCCAGATCAACCTCGGCTTCGGCTCCGACCCCAGCGCCAACATCAACGGCGGCCCTCCCAACATGA
GCAACCCCATGATCGCCCCCTTCCAGATCATCGGCCCCAGCAACAACCCCTACCCGGGCACCGTCTGCCTCA
CCCAGATCCCGCTCCCCGTCGGCGCCAAGGTCAAGGCCGGCGACGAGGCCACCATCCAGGTCGTCGAGAC
GGCCCAGCACGGCGCCGCCCTGTACTCGGTAGGCCTTGCTCCTCCCTCCTCCTTCTCCTCCTCCTCCTCC
CCCCACCATCTCCAGCGTACGACTAACCCGCAATCCAGTGCGTCGACATCATCTTCGCCGAGCAGGGCGACC
CCCGCATCGCCCCCGTCAACGAGTCCAACTGCTACAACTCGACAGACATTGGCTTCGCCCAAATCTACACCAT
CACCACCAAGGACTCGGGCTCCGACAACTACACCACCTCGGCCGCCCGGCGCACCCTCCACTCCGCGACCT
GGGTCGGCTGGACCCCCCTAGTATTTGCCGGCGTTGCCCTACTGCTGCTGTGA

> amino acid sequence encoded by *IcFBR1* gene in *Isaria cicadae*

MKLNLIISLALAGLAAAHTVISYPGWRGNNLITNDTFPYGMQWMYPCGGMGVTKNRTYWPIGGGTVAFQP
GWFRGHSLAFVQINLGFGSDPSANINGGPPNMSNPMIAPFQIIGPSNNPYPGTVCLTQIPLPVGAKVKAGDEA
TIQVVETAQHGAALYSCVDIIFAEQGDPRIAPVNESNCYNSTDIGFAQIYTITTKDSGSDNYTTSAARRTLHSATW
VGWTPLVFAGVALLLL
